# Higher thermal plasticity in flowering phenology increases flowering output

**DOI:** 10.1002/ece3.11657

**Published:** 2024-07-01

**Authors:** Aino Kotilainen, Anniina L. K. Mattila, Charlotte Møller, Susanna Koivusaari, Marko‐Tapio Hyvärinen, Maria H. Hällfors

**Affiliations:** ^1^ Botany and Mycology Unit, Finnish Museum of Natural History University of Helsinki Helsinki Finland; ^2^ Nature Solutions Unit Finnish Environment Institute (Syke) Helsinki Finland; ^3^ Research Centre for Environmental Change, Organismal and Evolutionary Biology Research Programme, Faculty of Biological and Environmental Sciences University of Helsinki Helsinki Finland; ^4^ Department of Geosciences and Geography University of Helsinki Helsinki Finland

**Keywords:** climate change, evolutionary ecology, flower, greenhouse experiment, *Hypericum*, phenotypic plasticity, reaction norm

## Abstract

Ongoing climate change poses an increasing threat to biodiversity. To avoid decline or extinction, species need to either adjust or adapt to new environmental conditions or track their climatic niches across space. In sessile organisms such as plants, phenotypic plasticity can help maintain fitness in variable and even novel environmental conditions and is therefore likely to play an important role in allowing them to survive climate change, particularly in the short term. Understanding a species' response to rising temperature is crucial for planning well‐targeted and cost‐effective conservation measures. We sampled seeds of three *Hypericum* species (*H. maculatum*, *H. montanum*, and *H. perforatum*), from a total of 23 populations originating from different parts of their native distribution areas in Europe. We grew them under four different temperature regimes in a greenhouse to simulate current and predicted future climatic conditions in the distribution areas. We measured flowering start, flower count, and subsequent seed weight, allowing us to study variations in the thermal plasticity of flowering phenology and its relation to fitness. Our results show that individuals flowered earlier with increasing temperature, while the degree of phenological plasticity varied among species. More specifically, the plasticity of *H. maculatum* varied depending on population origin, with individuals from the leading range edge being less plastic. Importantly, we show a positive relationship between higher plasticity and increased flower production, indicating adaptive phenological plasticity. The observed connection between plasticity and fitness supports the idea that plasticity may be adaptive. This study underlines the need for information on plasticity for predicting species' potential to thrive under global change and the need for studies on whether higher phenotypic plasticity is currently being selected as natural populations experience a rapidly changing climate.

## Graphical abstract

We studied the reproductive traits of three Hypericum species grown under four different temperature regimes to simulate current and predicted climatic conditions in their distribution ranges. We found a connection between plasticity and fitness, supporting the idea that plasticity may be adaptive. Ultimately, this study underlines the need for information on plasticity for predicting species' potential to thrive under global change.

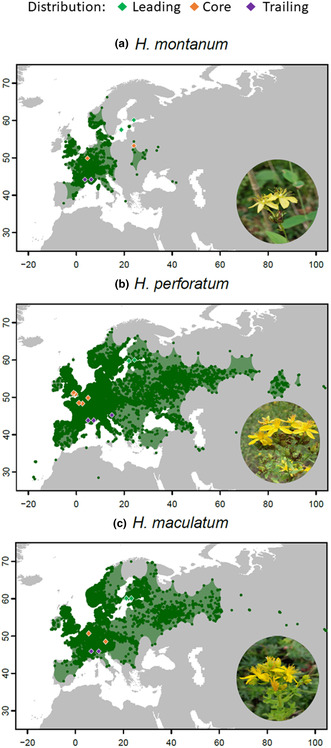

## 1 INTRODUCTION

The rapid rate of climate change may often outpace the ability of species to evolve genetically or disperse to new areas (Jump & Peñuelas, 2005; Radchuk et al., 2019). Therefore, phenotypic plasticity, which allows individuals to adapt to prevailing conditions, may be crucial for species to survive in a warming world. Phenotypic plasticity can act as a buffer and buy time to help species avoid extinction in the short‐term while they adapt genetically and disperse across space (Fox et al., 1999; Jump & Peñuelas, 2005; Matesanz & Ramírez‐Valiente, 2019; Nicotra et al., 2010). However, it remains unclear whether phenotypic plasticity alone will suffice for species to survive in changing environments.

The effects of climate change on species and their populations are manifold, and the most critical changes affecting species survival may be related to shifts in environmental conditions that act as cues for important life‐history events, such as the timing of flowering in plants (Piao et al., 2019). Flowering during the optimal time, when the likelihood of drought or frost is minimal, and pollinators are available, is imperative for a plant to successfully produce offspring. The timing of flowering is dictated by environmental conditions, mostly abiotic cues such as light and temperature (Wang et al., 2020), and thermal plasticity is an important way to respond to these abiotic phenological cues and changing temperatures. Optimal timing of flowering is important because of detrimental environmental conditions early in the season. Temperature is an important cue for the timing of life stages (Cook et al., 2012), but many species have also developed sensitivity to photoperiod and other environmental cues alongside temperature (Richardson et al., 2017; Wang et al., 2020).

As a result of climate change, the timing of thermal cues has advanced, thus advancing the start of the growing season (Arias et al., 2021). Not surprisingly, many studies have found a recent advance in phenological timing across an array of different species (Roslin et al., 2021; Zhang et al., 2015). However, not all species have advanced their phenology with climate change (Rosbakh et al., 2021; Zhang et al., 2015). This may be due to differential types of cues utilized, differential responses to the used cues, or simply due to a lower degree of plasticity to thermal cues specifically (Flynn & Wolkovich, 2018). Phenological responses may also vary among populations of the same species based on intraspecific local adaptation. For example, due to more severe and variable seasonal environments, range‐edge populations tend to exhibit greater phenotypic plasticity in general, thus being better adapted to climatic heterogeneity than populations at distributional core areas (Brancalion et al., 2018; Schmid et al., 2019; Usui et al., 2023). Furthermore, populations at the warmer distributional trailing edge may, in general, respond more strongly than those from the cooler leading edge (de Villemereuil et al., 2018; Richardson et al., 2017), though many studies have also come to contrasting conclusions, as pointed out in the review by Zettlemoyer and Peterson (2021). More specifically, species from warmer and more stable environments could have higher thermal sensitivity to flowering phenology (Zhang et al., 2015). However, greater phenotypic plasticity is not necessarily equivalent to higher trait averages. Core distribution areas are often located at the most optimal conditions within a species range and due to these optimal conditions, it is reasonable to assume that at the core distribution areas, species have optimal performance and the highest averages of related traits (Halbritter et al., 2015). However, due to climatic changes, core distribution areas might be shifting, and it is therefore imperative to investigate the relationship between plasticity and trait averages across all distribution areas of a species to improve predictions of species distribution and their potential niche tracking.

Here, we study thermal plasticity in flowering phenology in three *Hypericum* species, and whether plasticity varies among species or populations originating from different parts of their distribution areas. We hypothesized that populations from the distributional edges are more plastic than populations from the core areas, but that core area populations may have higher trait values on average. Furthermore, in *H. perforatum*, for which we also collected fitness data, we investigate the novel relationship between thermal plasticity and fitness (Iler et al., 2021) to test more specifically whether thermal plasticity in flowering phenology is connected to plant fitness.

## 2 MATERIALS AND METHODS

### 2.1 Study species and populations

**FIGURE 1:**
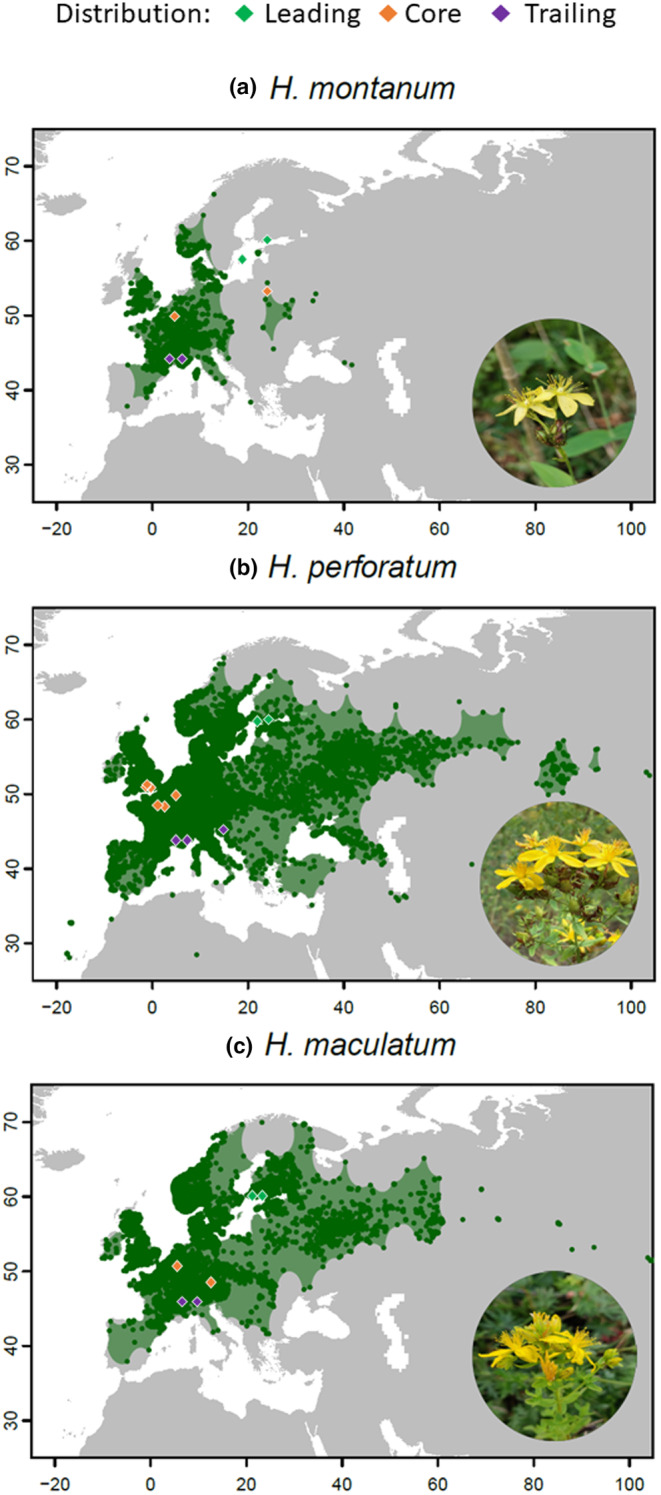
Distribution areas of (a) *Hypericum montanum*, (b) *H. perforatum*, and (c) *H. maculatum* during years 1900–2022. Dark green points are filtered observations of the species, while the lighter green polygon represents the projected distribution based on observations published in GBIF (www.gbif.org). Origins of the study populations are indicated with different colors representing their distribution: Leading (green), core (orange), and trailing (purple). Photo credit of *H. montanum*: https://www.inaturalist.org/observations/88095325, *H. perforatum*: https://www.inaturalist.org/observations/172389923, and *H. maculatum*: https://inaturalist.ca/observations/53887149.

*Hypericum maculatum* (Crantz), *H. montanum* (L.), and *H. perforatum* (L.) are perennial herbs native to Europe. They belong to the *Hypericaceae* family and share many characteristics, such as yellow flowers and leaf arrangement. *H. perforatum* is facultatively apomictic and can reproduce successfully by self‐fertilization. It also has a high capacity for producing seeds (Crompton et al., 1988). *H. maculatum* and *H. montanum* on the other hand are obligate sexual reproducers (Matzk et al., 2003). The native ranges of *H. maculatum* and *H. perforatum* extend from southern to Northern Europe (Figure 1) and they are commonly found in grassland habitats (Hypericum maculatum Crantz in GBIF Secretariat, 2022; Hypericum perforatum L. in GBIF Secretariat, 2022). In comparison, the former has a more restricted distribution than the latter (Figure 1). At last, *H. montanum* has the narrowest distribution range out of our three study species, especially at higher latitudes (Figure 1), and is a habitat specialist occurring more scarcely and mainly in woodlands (Hypericum montanum L. in GBIF Secretariat, 2022).

To map the distribution areas of the three *Hypericum* species (Figure 1), we downloaded their occurrence records from GBIF (www.gbif.org) on July 21, 2023. We filtered the records by removing records lacking coordinate information and were located in water areas, and only kept the records if they had an existing institution code, were made between the years 1900–2022, had coordinate uncertainty less than or equal to 21,000 m, and presented the current occurrence status. Furthermore, we only incorporated one record per species per location as well as one record per 21 km^2^ grid cell. We used the *getDynamicAlphaHull* function from the *RangeBuilder* package (Davis Rabosky et al., 2016) to generate distribution polygons for the three *Hypericum* species. The polygons were set to enclose a minimum of 80% of sightings. The alpha value was set to 3 (Burgman & Fox, 2003), and a maximum of 10 disjunct polygons were allowed. The buffer was set to ±21,000 to match the coordinate accuracy from GBIF. The polygons were set to cover only terrestrial areas.

We acquired seeds from various locations within the European distribution ranges of the study species, contingent upon their availability. This was done to encompass the trailing (southern distributional edge), core, and leading (northern distributional edge) areas of the species distribution. We chose seed accessions, i.e., seeds collected from the same location at the same time, among the accessions available in managed seed banks (e.g., Millennium Seed Bank, The European Native Seed Conservation Network ENSCONET partners (Eastwood & Rivière, 2009)) and augmented them with populations collected afresh. In total, we included 23 populations in the experiment: six *H. maculatum*, six *H. montanum*, and 11 *H. perforatum* populations (Table 1). The seed material from managed seed banks were pooled samples collected according to ENSCONET guidelines (generally originating from at least 50 individuals; ENSCONET, 2009). The seeds that we collected ourselves were sampled per mother individual, and an equal number of seeds from each mother individual (averages 24 mother individuals per accession) were pooled in each treatment.

**TABLE 1:** The study populations and their localities.

Species	Distribution	Collected	Country	Latitude	Longitude	Origin	No. of mothers
*H. maculatum*	L	2020	Finland	60.18	24.70	Self‐collected	25
*H. maculatum*	L	2020	Finland	60.26	23.60	Self‐collected	25
*H. maculatum*	C	2020	Belgium	50.50	6.25	Self‐collected	25
*H. maculatum*	C	2018	Austria	48.11	13.29	Seedbank	‐
*H. maculatum*	T	2021	France	46.14	6.59	Self‐collected	21
*H. maculatum*	T	2008	Italy	46.12	9.57	Seedbank	‐
*H. montanum*	L	2020	Sweden	57.79	18.89	Self‐collected	25
*H. montanum*	L	2021	Finland	60^a^	24^a^	Self‐collected	25
*H. montanum*	C	2017	Poland	52.70	23.85	Seedbank	‐
*H. montanum*	C	2021	Belgium	50.09	4.57	Self‐collected	19
*H. montanum*	T	2021	France	44.42	5.46	Self‐collected	25
*H. montanum*	T	2019	France	44.18	3.43	Seedbank	‐
*H. perforatum*	L	2021	Finland	60.21	24.96	Self‐collected	25
*H. perforatum*	L	2021	Finland	59.83	22.93	Self‐collected	25
*H. perforatum*	C	2021	France	48.40	2.63	Self‐collected	15
*H. perforatum*	C	2021	France	48.83	2.11	Self‐collected	25
*H. perforatum*	C	2021	Belgium	50.08	4.55	Self‐collected	25
*H. perforatum*	C	2021	UK	50.90	−0.04	Self‐collected	25
*H. perforatum*	C	2021	UK	50.88	−0.02	Self‐collected	25
*H. perforatum*	C	2021	UK	50.87	−0.00	Self‐collected	25
*H. perforatum*	T	2021	France	44.76	6.28	Self‐collected	25
*H. perforatum*	T	2021	France	44.45	5.44	Self‐collected	23
*H. perforatum*	T	2020	Slovenia	45.64	14.23	Seedbank	‐

*Note*: Distribution areas are denoted as L = leading, C = core, and T = trailing area.

^a^ The exact location could not be disclosed due to conservation status (CR; Hyvärinen et al., 2019). Number of mothers were unknown for populations originating from seedbanks.

### 2.2 Temperature treatments and greenhouse cultivation

We grew the plants under common garden conditions in greenhouses of the Viikki Plant Growth Facilities, University of Helsinki, from December 2021 to May 2022. In May, we transferred the plants outside for seed maturation. We collected seeds from the experimental individuals during summer and weighed them from August to October 2022.

In the greenhouses, we grew the plants under four different temperature treatments with two replicates for each, totaling eight greenhouse compartments, and each species on a separate table within each compartment. The daytime (16 h) temperatures were set to 16°C (Cold), 20°C (Medium), 24°C (Warm), and 28°C (Hot). The choice of temperature treatments was loosely based on data on average summer temperatures at the trailing‐, core‐, and leading distributional areas of the study species from the climatic information service WorldClim (https://www.worldclim.org). The night‐time temperatures (8 h) were set at 8°C and 10°C below the daytime temperature at the germination and vegetative stages, respectively. Photoperiod in all treatments was 16/8 h light/dark. In addition to the automated temperature settings, we monitored realized temperature conditions at the plant level using temperature loggers (Lascar EL‐USB‐2‐LCD+). The realized temperatures were somewhat higher than the set temperatures, particularly as solar radiation increased with the advancing spring, but the differences between treatments remained approximately equal (10.5061/dryad.f4qrfj73k).

All seeds were cold stratified for 4 weeks at 4°C in dry paper bags. On December 1, 2021, we sowed 25 seeds per population and replicate them into trays filled with the sowing mixture (Kekkilä kylvöseos W HS R8017; KEK31116) with seeds of two populations sown on each tray separated by a border of cardboard and sand. We then placed the trays on a water retaining rug on greenhouse growing tables and its soil topped with coarse sand after sowing the seeds. In the beginning of February 2022, we randomly chose up to 10 (depending on availability) of the germinated plants per population, treatment, and replicate to be included in the experiment and transferred them from trays to individual 1 L pots filled with soil (Kekkilä Professional Karkea Ruukutusseos; KEK33933) and we then placed the pots on a water retaining rug on greenhouse growing tables. At the same time, we propped up the plants on a support stick if the plant was large enough. We marked all plant individuals with QR‐coded ID tags. We separated the individuals of each population into replicates A and B, each with up to 10 plants. To avoid microclimatic biases, we periodically rotated both the germination trays and later the plant pots (dates of rotation: Dec 8, 2021, Dec 15, 2021, Dec 22, 2021, Jan 19, 2022, Jan 26, 2022, Feb 2, 2022, Mar 4, 2022, Apr 8, 2022). We regularly fertilized the plants with Kekkilä turve superex fertilizer solution; 0.075% solution was applied on Jan 14, 2022, and 0.2% solution was applied on Apr 1, Apr 4, and Apr 29, 2022.

Watering was implemented by an automated watering system with treatment‐specific schedules to keep all experimental plants equally moist. The watering system of *H. perforatum* in the “Hot”‐treatment, replicate A, broke at the end of March leaving the plants dry for some days, which we take into account in the interpretation of the results.

### 2.3 Data collection

Starting 2 months after sowing, we collected flowering phenology data by monitoring the plants two times per week and recording the date of the first observation of an open flower for each plant individual.

During a four‐week data collection phase at peak flowering (April–May), we counted flowers at different stages (i.e., bud, flowering, flowered, seed capsule), except for *H. maculatum* and *H. montanum* populations which could not be counted in the “Medium” and “Cold” treatments due to limited time. For each study individual, up to five seed capsules or withered flowers were marked to ensure a matching collection of seeds after ripening. After the flowering counts, we transferred the plants to outdoor conditions at the Kumpula Botanic Garden to allow the seeds to ripen over the summer, whereafter we collected them. We dried the collected seeds for a minimum of 5 days in RH 15% and cleaned them using sieves (800 μm and 250 μm), after which we counted and weighed them. We used average seed mass (total seed mass/total seed number) as a measure of reproductive output, as some of the seeds could have dispersed before the capsules were collected, rendering the total seed number an unreliable proxy measure of fitness.

### 2.4 Data analyses

To test for the effect of distribution area (leading, core, and trailing) on plasticity in flowering phenology, we ran linear mixed‐effect models (LMMs) in R version 4.2.2 (R Core Team, 2022) using the *lmer* function of the *lme4* package (Bates et al., 2015). We applied linear mixed effects models to explain how temperature treatment and distribution area affected variation in flowering phenology. For each species, we used the day of the year of first flowering (“DOY”; i.e., Julian day, continuous variable with assumed normally distributed residuals) as the response variable, and the temperature treatment, region, and their interaction as explanatory variables. Population and replicate were included in the model as random effects. Because there was a linear relationship between flowering time and temperature, it was possible to convert temperature into a continuous variable (day‐time temperature). This allowed us to obtain an estimate of the effect of treatment temperature on the DOY of flowering, interpreted as a population‐specific measure of plasticity (i.e., the reaction norm). Furthermore, a species comparison was performed using flowering start as the response variable, the interaction between temperature treatment and species as explanatory variables and replicate nested within the distribution area as a random effect. This allows us to account for the variation introduced by replicate. A post hoc Tukey test was applied to disentangle significant species differences. Finally, predictions of flowering timing were obtained via the function *predict* on a modified data set ranging from the minimum to maximum temperature values and accounting for all levels of categorical variables included.

Second, to assess the contribution of each covariate to our model fits, we used Akaike's Information Criterion (AIC; Burnham & Anderson, 2002) in a stepwise model selection method. We fitted a model with no explanatory variables as a baseline (a so‐called intercept‐only model), as well as models with either temperature or region or both with and without an interaction effect. If the difference in the goodness‐of‐fit between the two models exceeded an absolute value of 2 (Vrieze, 2012), we interpret the parameter included in the model with the lowest AIC value as significantly increasing the model fit. The most parsimonious model was used for further downstream analyses.

Third, we used population‐level data of *H. perforatum* to test for a connection between plasticity and fitness. To retrieve population‐level estimates of fitness traits (flower count and average seed mass), the trait values were averaged first inside treatments and then across all treatments within each population. To allow comparison of fitness proxies (measured as the slope of the linear relationship between the day of flowering and temperature for each population, the models including temperature treatment as an explanatory variable and replicate as a random effect). We then modeled the effect of the slope value on fitness proxies using the *lm* function in R.

Finally, to differentiate the effects of plasticity from that of earlier flowering (and thus longer flowering time during which the individual could have time to produce more flowers) on flower count, we removed the linear effect of flowering time on flower count. To do this, we modeled how the duration of flowering (the number of days the plant had been in the flowering stage before the flower count) affected the logarithm of flower abundance, including population and replicated as random effects. Residuals of this model were then used as explanatory variables in a model explaining the effect of the residuals on plasticity, with distribution area and the interaction between temperature treatment and distribution area as additional variables.

For all models, the Shapiro–Wilk test was used to assess whether the assumptions of normality of model residuals were met. Similarly, the Bartlett test was used to assess whether the assumptions of homoscedasticity of model residuals were met.

## 3 RESULTS

We measured flower phenology on a total of 904 individuals of the three different study species, grown under four temperature treatments. The individuals originated from 23 populations from three distribution areas.

**FIGURE 2:**
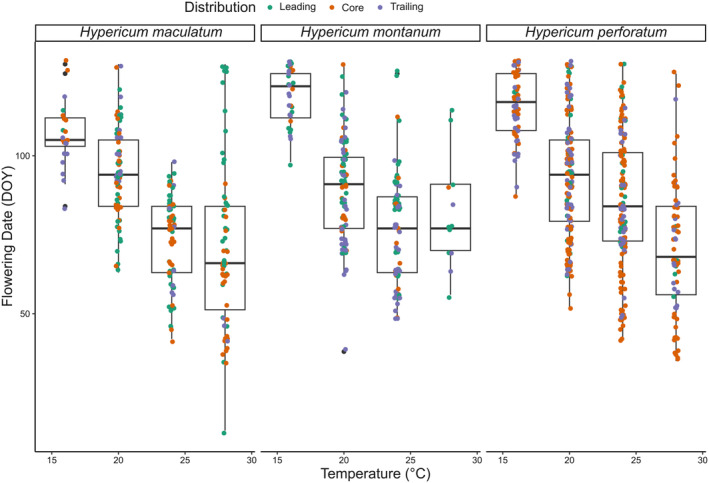
Date of flowering (DOY) as a function of temperature grouped by species and distribution area. The distribution area is indicated by color: Leading (green), core (orange) and trailing (purple). Horizontal jitter was introduced to aid visualization.

**FIGURE 3:**
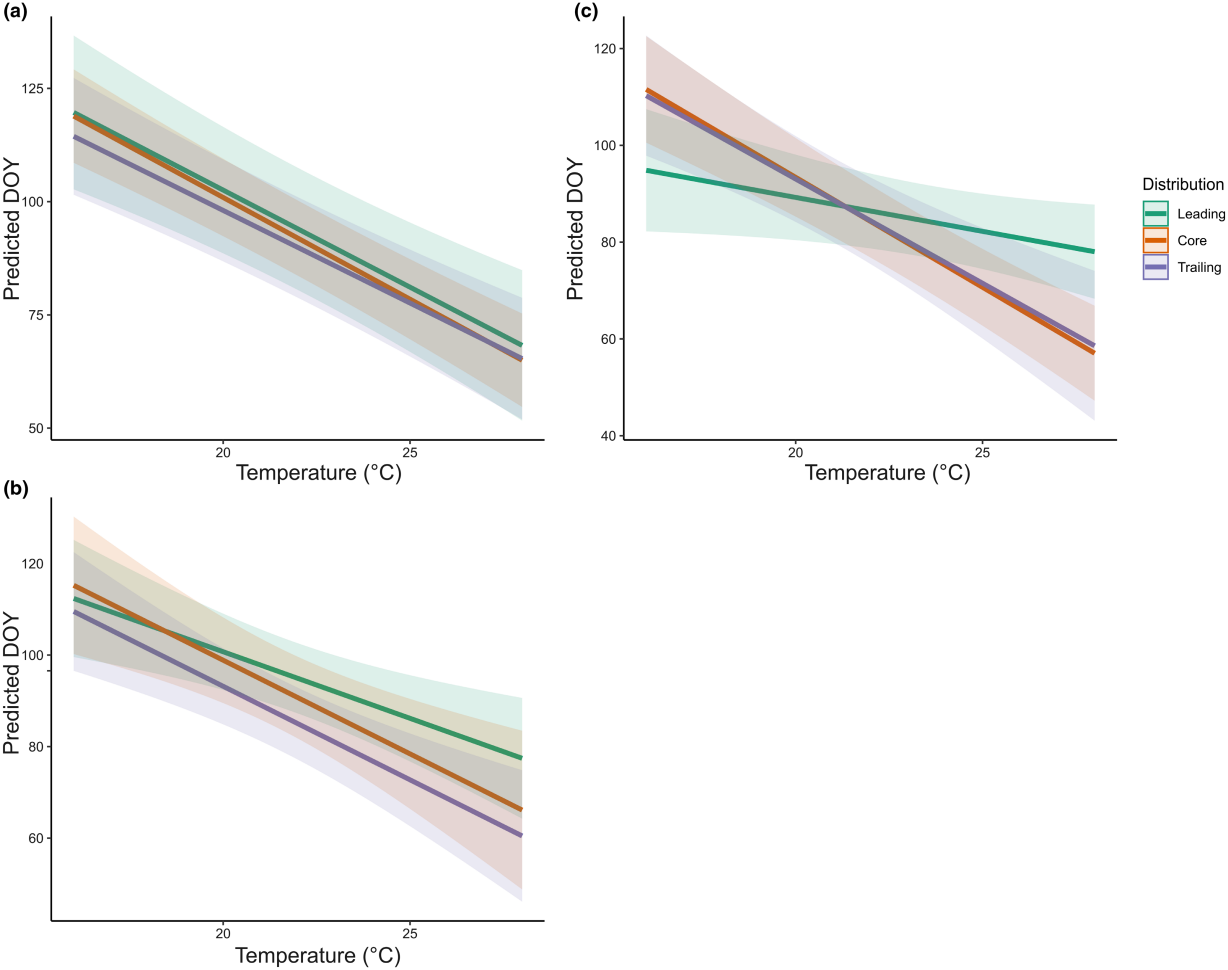
Predicted flowering day (DOY) as a function of temperature per the seed sampling area (green = leading; orange = Core; purple = Trailing) for (a) *Hypericum perforatum*, (b) *H. montanum*, and (c) *H. maculatum*. The shaded area shows confidence intervals of predictions.

We found that individuals of all three species flowered earlier in warmer temperatures (Figures 2 and 3). Species comparisons revealed highly significant effects of treatment, species, and their interaction on the flowering start (Table 2). A post hoc Tukey test revealed that *H. maculatum* flowered significantly earlier than *H. perforatum* (*p* = .001), while there was only weak evidence of a difference in flowering start between *H. maculatum* and *H. montanum* (*p* = .098), and *H. montanum* and *H. perforatum* did not differ from each other (*p* = .730).

**TABLE 2:** Results of mixed‐effects model investigating the effect of treatment, species, and their interaction on flowering start.

	Chisq	df	*p*
Treatment	261.269	3	<.001
Species	17.831	2	<.001
Treatment: Species	25.493	6	<.001

*Note*: Replicate was nested within distribution area and added as a random effect.

Earlier flowering at warmer temperatures was also revealed by model comparison using AIC, where temperature increased model fit for *H. perforatum* and *H. montanum* as compared to the intercept‐only model (Table 3; Figure 3) with an estimated advance in flowering by 4.29 and 2.91 days per°C, respectively (Table 4). For *H. maculatum* we found weak evidence (*p* = .062) of a temperature effect with an estimated 1.4 day advance per increased°C.

**TABLE 3:** AIC comparisons of models testing the variables temperature treatment, distribution area, as well as their interaction, against the flowering start for the three study species *Hypericum perforatum, H. maculatum*, and *H. montanum*.

	*H. perforatum*			*H. maculatum*			*H. montanum*		
	AIC	Δdf	*p*‐Value	AIC	Δdf	*p*‐Value	AIC	Δdf	*p*‐Value
Intercept‐only	3974.3			2016.2			1786.0		
Temperature	3956.1	1	<.001	2000.5	1	<.001	1777.9	1	.002
Distribution area	3977.9	1	1.000	2016.8	1	1.000	1781.5	1	1.000
Temperature + Distribution area	3959.7	1	<.001	2000.2	1	<.001	1773.4	1	.001
Temperature × Distribution area	3963.0	2	.699	1983.8	2	<.001	1774.4	2	.220

*Note*: Akaike's Information Criterion (AIC), the difference in the degrees of freedom (Δdf), and *p*‐value are provided.

**TABLE 4:** Summary statistics of models explaining the timing of flowering with temperature, distribution area, and their interaction (based on the most parsimonious model temperature × distribution area from Table 3) separately for the three study species: *Hypericum perforatum*, *Hypericum maculatum*, and *Hypericum montanum*.

	*H. perforatum*				*H. maculatum*				*H. montanum*			
	Estimate	SE	*t*‐Value	*p*‐Value	Estimate	SE	*t*‐Value	*p*‐Value	Estimate	SE	*t*‐Value	*p*‐Value
Intercept	188.27	20.09	9.37	<.001	117.27	16.77	6.99	<.001	158.96	19.90	7.99	<.001
Temperature	−4.29	0.85	−5.04	<.001	−1.40	0.70	−2.01	.062	−2.91	0.89	−3.26	.011
Core	2.48	18.68	0.13	.894	66.97	16.75	4.00	<.001	21.80	20.35	1.07	.285
Trailing	−8.43	20.03	−0.42	.674	61.91	22.37	2.77	.006	16.01	15.85	1.01	.315
Temperature × Core	−0.21	0.77	−0.27	.790	−3.14	0.68	−4.61	<.001	−1.18	0.95	−1.25	.213
Temperature × Trailing	0.20	0.83	0.24	.811	−2.91	1.00	−2.90	.004	−1.18	0.73	−1.62	.108

*Note*: Estimates, standard errors (SE), *t*‐values and *p*‐values are provided.

The most parsimonious model was studied, and according to AIC, neither including the distribution area nor its interaction with temperature improved model fit in *H. perforatum* (Table 3). In *H. maculatum*, the distribution area helped explain variation in the temperature response of flowering phenology, as indicated by an improved model fit when including an interaction term between distribution area and temperature (Table 3). In *H. montanum*, the distribution area, but not the interaction term, increased model fit, indicating that the distribution areas differed in average flowering times (DOY_Leading_ = 91.66, DOY_Core_ = 89.65, DOY_Trailing_ = 83.60) but not in their responses of flowering time to temperature (Table 3).

In *H. maculatum*, the leading‐edge populations differed from the trailing and core populations, showing both a different mean phenology and response norm. The leading‐edge populations flowered on average 2 days earlier than the populations from the trailing and core areas. Furthermore, at colder temperatures, leading‐edge populations of *H. maculatum* were predicted to flower 13.5 and 16.5 days earlier than core and trailing‐edge populations, respectively. The leading populations were also less plastic, responding with an advance of 1.4 days per°C, while the core and trailing populations responded with an advance of 4.54 and 4.05 days/°C, respectively.

**FIGURE 4:**
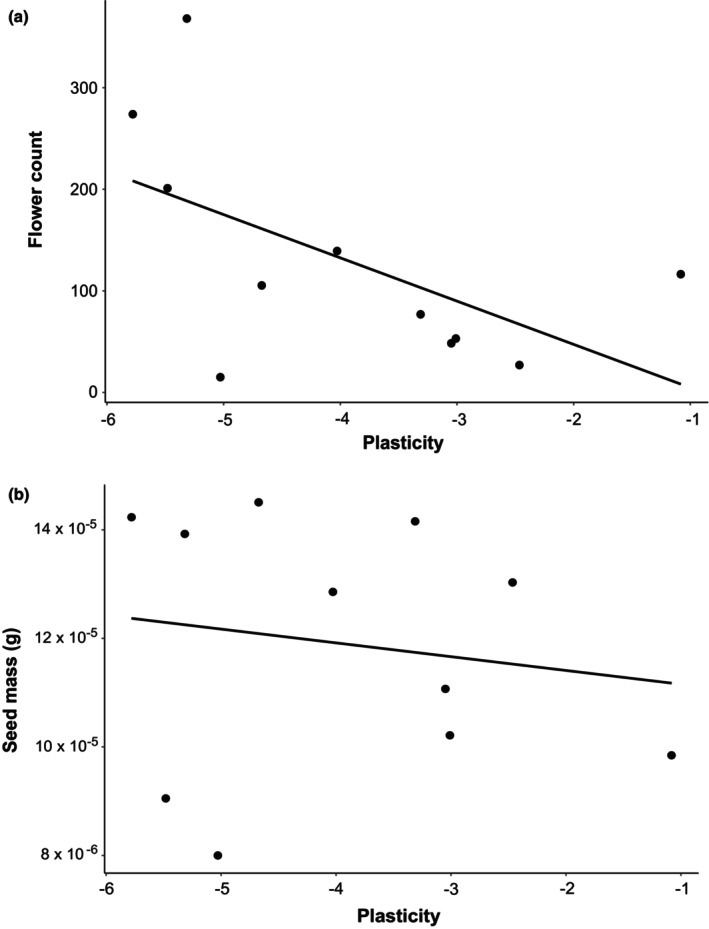
Flower count (a) and seed mass (b) as a function of plasticity. Each point represents one *H. perforatum* population. Plasticity was measured as day of year of flowering as a response to temperature, meaning that more negative values indicate greater plasticity in phenology.

**FIGURE 5:**
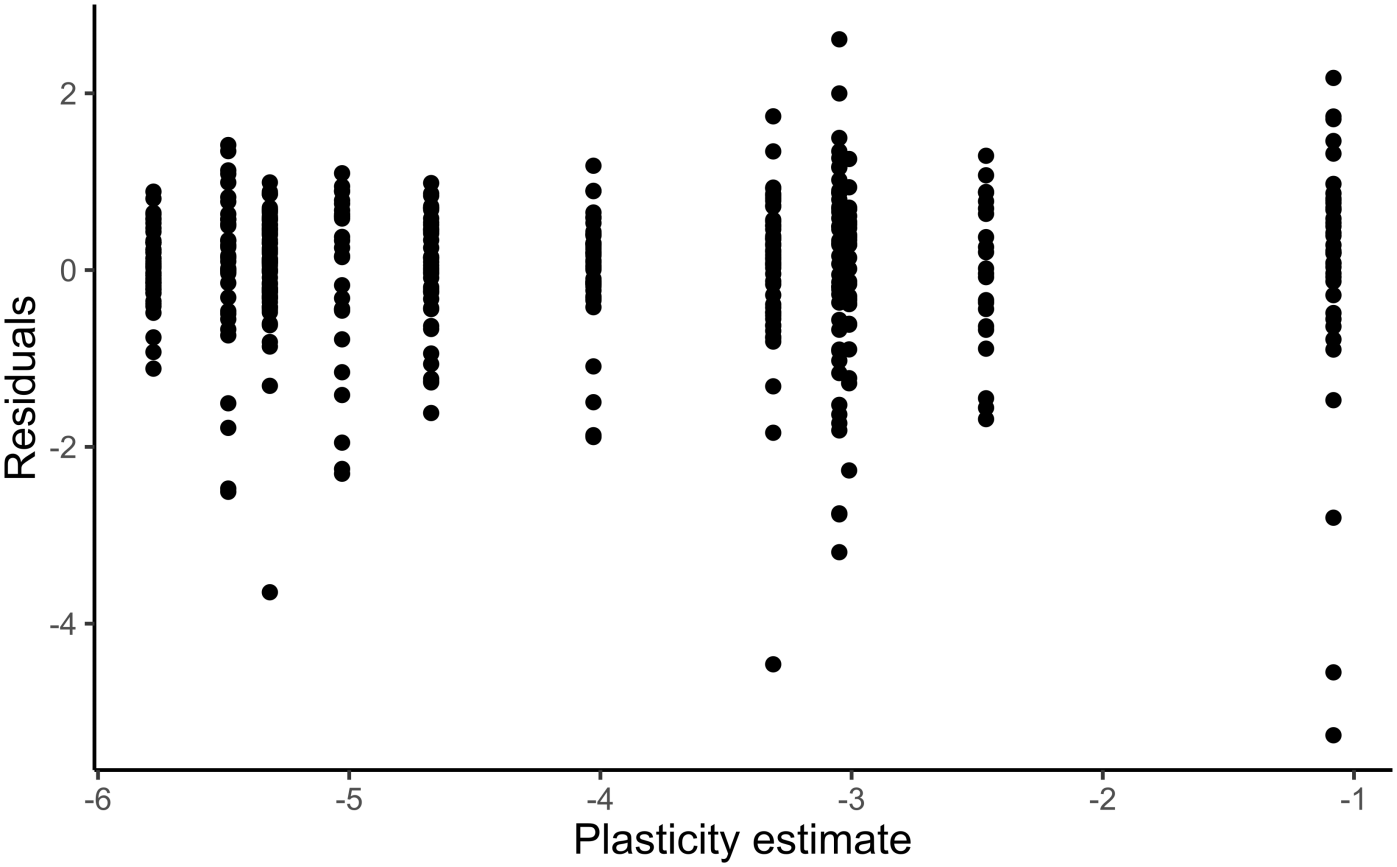
Model residuals derived from the linear effect of flower longevity on flower count (log) as a function of *Plasticity* and *Distribution group*, plotted against the plasticity estimate.

There seemed to be a tendency for plasticity (derived from the estimate of the slope in the model testing the effect of temperature treatment on the date of flowering) to have a positive relationship with flower abundance (*p* = .070, Table 5; Figure 4a). Plasticity in flowering time did not influence the mass of individual seeds produced (Table 5; Figure 4b). The effect on flower abundance was consistent across treatments, indicating that temperature itself did not have a confounding effect on plasticity estimates through, e.g., the individuals from plastic populations flowering earlier and producing more flowers only in the warmest treatments. Instead, plastic populations tended to produce more flowers in all treatments (Figure 4a). The residual model, however, revealed that this was likely an effect of plastic individuals being able to flower early and thus having more time to produce flowers by the time of counting, as the number of days in flower captured a large share of the explainable variation with the residuals having no explanatory power for flower count (Table 6; Figure 5). Thereby, individuals from plastic populations started flowering earlier and thus had had time to flower for longer and thus develop more flowers until the time of recording the flower count.

**TABLE 5:** Models testing the effect of plasticity on traits indicating reproductive fitness: Flower count and seed mass in *H. perforatum*.

	Estimate	SE	*t*‐Value	*p*‐Value
Flower count				
Intercept	−19.66	77.99	−0.25	.807
Plasticity	−39.03	19.00	−2.05	.070
Seed mass				
Intercept	1.09e‐04	2.18e‐05	5.01	<.001
Plasticity	−2.54e‐06	5.22e‐06	−0.49	.638

*Note*: Plasticity is measured as slope of the linear relationship between flowering time and temperature, with negative values indicating greater plasticity.

**TABLE 6:** Model residuals derived from the linear effect of flower longevity on flower count (log) as a function of *Plasticity* and *Distribution group*.

	Estimate	SE	*t*‐Value	*p*‐Value
Intercept	0.08	0.48	0.16	.870
Plasticity	0.03	0.12	0.26	.798
Core	−0.10	0.50	−0.20	.844
Trailing	−0.16	0.60	−0.27	.789
Plasticity: Core	−0.04	0.12	−0.31	.757
Plasticity: Trailing	−0.05	0.14	−0.33	.740

## 4 DISCUSSION

Our common garden study, employing three *Hypericum* species sourced from diverse distribution areas and populations and subjected to varying temperature treatments, unveiled significant and species‐specific responses in plasticity towards flowering initiation amidst rising temperatures. We also found variation in the thermal plasticity of flowering phenology among trailing‐, core, and leading‐edge populations. Finally, focusing on fitness consequences in *H. perforatum*, our results indicated that higher plasticity in flowering phenology may lead to increased fitness.

### 4.1 Flowering phenology tracks temperature

Our results show that, in the three studied *Hypericum* species, flowering phenology is a plastic trait that responds by advancing in seasonal time as temperatures rise, consistent with some previous studies (e.g., Haggerty & Galloway, 2011; Matthews & Mazer, 2016; Richardson et al., 2017; Roslin et al., 2021; Zhang et al., 2015). Several experimental studies using a common garden have also demonstrated similar results (de Villemereuil et al., 2018; Haggerty & Galloway, 2011), indicating that the observed phenological advances in natural populations are at least in part due to phenotypic plasticity. However, this study contributes valuable information on inter‐specific phenological differences between generalist and specialist species occurring across Europe.

### 4.2 Climatic variation across species' ranges can cause intraspecific differences in phenological responses

Although the general pattern in flowering phenology was similar among the study species, for *H. maculatum* and *H. montanum* there were clear differences in how the flowering phenology of populations from the different parts of distribution areas responded to temperature. One could speculate that stronger gene flow between populations in *H. perforatum*, a more common habitat generalist species (Tero et al., 2003), may prevent local adaptation of populations and explain similar plastic responses along the species' distributional range.

In *H. maculatum*, the leading area populations were less plastic in their response to temperature compared to the other population origins. Individuals from the leading area tended to flower earlier in cold conditions but later in warm conditions compared to the individuals from other origins. Contrary to our expectations, this indicates less sensitivity to temperature in the leading range edge, where the climate tends to be cooler, and the spring season starts later in the year. The relatively earlier flowering in cold conditions of plants originating from a cooler climate is likely an adaptation to the shorter growing season, under which the plants must capitalize on the few warm months for their reproduction. Thus, flowering earlier can enable them to finish producing fruit before the next winter.

For *H. montanum*, we found a difference only in the mean flowering time of trailing and leading areas but not among the slopes. In other words, there was no difference in plastic responses between the populations from different areas, but on average, flowering took place at different times. Overall, the trailing populations flowered earlier, leading populations later, and the core populations somewhere in between. This response is similar to the one observed for *H. maculatum*, except for under colder temperatures where leading populations from *H. maculatum* flowered earlier. Applicable for all three study species is that the trailing populations typically originate from a warmer climate where summer conditions arrive earlier than in the more northern areas and where a rapid response through flowering in these optimal conditions could be advantageous. In colder areas, early‐summer temperatures can be unpredictable, and thus a more conservative response can save the plants from flowering too early (Wang et al., 2014; Zhang et al., 2015).

Though contradicting our hypotheses, our results are consistent with previous studies showing that plants growing at higher latitudes (Richardson et al., 2017) and altitudes (de Villemereuil et al., 2018) tend to have less phenological plasticity. This could be attributed to plant stress responses (de Villemereuil et al., 2018), or be an indication of a more conservative strategy related to unpredictable weather conditions, whereby plants at higher latitudes may rely more heavily on photoperiod as an additional cue to time life‐history events (Richardson et al., 2017).

### 4.3 Plasticity of phenology can affect reproductive success

Few studies have previously linked phenology to traits relevant to fitness (however, see Iler et al., 2021). Here, we further focused on the effect of plasticity on fitness in one of the species, *H. perforatum*. Our results showed that more plastic populations tended to produce more flowers in all treatments. Accounting for the duration of flowering revealed that the higher number of flowers was likely caused by more plastic individuals having had more time to produce flowers until the time of counting. Thus, plasticity in itself did not lead to a higher reproductive output, but plastic populations were able to start flowering earlier and consequently secure a longer flowering period and, potentially, more time for seed ripening. Whether this equals a higher reproductive output by the end of the growing season, and thus gives them an advantage under future climatic conditions, would warrant further studies on reproductive output across the growing season. In theory, the later flowering plants (like *H. perforatum* flowering from July to September in Finland) could catch up in their reproductive output if they also continue flowering later into the season.

In a study on *Campanulastrum americanum*, Haggerty and Galloway (2011) found that plants that started flowering earlier had a more compact reproductive period, meaning that they ended their flowering sooner as well as ripened their fruit more rapidly. On the other hand, Zhang et al. (2015) found, in their multispecies study, that the start of flowering did not usually affect the length of the flowering period. Thus, the responses are likely to be species‐dependent, and here we can only speculate what this may mean for *H. perforatum*. A better understanding of how the flowering period of *Hypericum* species is affected by changes in flower phenology could help better understand the effects of phenological plasticity on fitness.

The number of flowers is only one of the factors determining the lifetime reproductive success (LRS) of a plant individual. The number and quality of seeds produced from those flowers are another fundamental fitness determinants as well as success in fertilization via pollen. Furthermore, low‐quality seeds may have a lower germination rate and lead to fewer and lower‐quality offspring. Here, we used individual seed mass as a proxy for seed quality. We showed that seed mass was not affected by phenological plasticity, indicating that plasticity, and thus an earlier start of flowering, was not related to the quality of the produced seeds. To assess ultimate fitness consequences, and thus gain a better understanding of selection acting on thermal plasticity of flowering phenology, future studies could measure seed count, as well as test the seed quality more directly by, e.g., germinating the seeds.

### 4.4 Thermal plasticity and adaptation of phenological traits in a changing climate

If phenological plasticity is advantageous in a warming climate, less plastic species and populations could be at a disadvantage. It seems that the inability to track an optimal seasonal environment through flowering phenology may affect species' fitness negatively (Cleland et al., 2012). While genetic variation benefits species survival, intra‐specific differences in phenological plasticity may lead to more complex outcomes for species viability. According to our results, leading‐edge population seem to be less thermally plastic, what consequences could this have for species adapting to climate change? Ultimately, leading edge populations could rely more on genetic adaptation and thus respond more slowly to the changing environment, leaving them at a potential disadvantage in responding appropriately to changing climatic conditions.

Plasticity and genetic adaptation are both means for a species to adapt to changing conditions. However, in nature, populations and species also have the option to disperse to new areas and track suitable climates across space when not limited by dispersal barriers or low dispersal rates. Intra‐range dispersal can also influence the gene pools of populations and thus, their adaptive potentials (Christmas et al., 2016; Jump & Peñuelas, 2005). Moreover, phenotypic plasticity itself may be subject to selection and encompass a diversity of environmentally induced responses leading to different evolutionary outcomes (Jump & Peñuelas, 2005; Ghalambor et al. 2007). Furthermore, phenotypic plasticity can affect the selective pressures of traits, and to this end, influence their patterns of adaptation (Christmas et al., 2016). In plastic traits, short‐term adjustments can happen through nongenetic means, and thus highly plastic traits may be under lower selective pressure, consequently leading to slower evolution in these traits (Grenier et al., 2016; Oostra et al., 2018).

### 4.5 Study limitations

The relatively low number of populations represented within each distribution area for each species in this study may somewhat limit the conclusions that can be drawn regarding the differences between areas. However, as our overall sample sizes covering several species of Hypericum with distinct distribution ranges are sufficient, the general trends observed in our data can still provide important building blocks for future studies.

Unfortunately, due to time constraints, flower counts and overall seed weight per individual could only be obtained for one species, and we therefore chose to direct our efforts to *H. perforatum*, as this is the most widely studied species so far due to its importance for the medical industry (Rizzo et al., 2020; Saddiqe et al., 2010).

## 5 CONCLUSION

A central question in climate change ecology is whether the phenological tracking of climate through plasticity, adaptation, and dispersal is and will be sufficient to keep up with the temperature change. Plasticity alone may not be enough, and it is likely that genetic adaptation will in addition be needed for a sufficient change in phenology (Anderson et al., 2012). Furthermore, other factors besides phenology could become more limiting for the species' future capacity to adjust as climate changes. Accounting for several modes of adjustment to climate change (Hällfors et al., 2021) as well as their interactions can make predicting future responses very complicated. As this study further highlights, intraspecific differences can have implications for fitness and processes related to surviving and producing viable offspring in a changing world. Populations of species may differ in their capacity to respond to global change by plastic and adaptive responses, and understanding such patterns will be important in predicting and facilitating the future survival of species.

## AUTHOR CONTRIBUTIONS

**Aino Kotilainen:** Data curation (equal); formal analysis (equal); investigation (equal); visualization (lead); writing – original draft (lead); writing – review and editing (lead). **Anniina L. K. Mattila:** Data curation (equal); formal analysis (equal); investigation (equal); project administration (equal); supervision (equal); visualization (supporting); writing – review and editing (supporting). **Charlotte Møller:** Formal analysis (supporting); visualization (supporting); writing – review and editing (equal). **Susanna Koivusaari:** Data curation (supporting); visualization (supporting); writing – original draft (supporting); writing – review and editing (supporting). **Marko‐Tapio Hyvärinen:** Conceptualization (equal); investigation (equal); project administration (equal); supervision (equal); writing – original draft (supporting); writing – review and editing (supporting). **Maria H. Hällfors:** Conceptualization (equal); data curation (equal); investigation (equal); methodology (equal); supervision (equal); visualization (supporting); writing – original draft (supporting); writing – review and editing (supporting).

## CONFLICT OF INTEREST STATEMENT

The authors have no conflict of interest to declare.

## Data Availability

All data and code used to conduct the analyses, as well as greenhouse temperature conditions and detailed watering schedule, are available at the Dryad Digital Repository: DOI: 10.5061/dryad.f4qrfj73k.
